# Perceived Teacher Autonomy Support and Students' Deep Learning: The Mediating Role of Self-Efficacy and the Moderating Role of Perceived Peer Support

**DOI:** 10.3389/fpsyg.2021.652796

**Published:** 2021-06-21

**Authors:** Jingxian Zhao, Yue Qin

**Affiliations:** Faculty of Education, Languages & Psychology, SEGi University, Kota Damansara, Malaysia

**Keywords:** perceived teacher autonomy support, self-efficacy, perceived peer support, deep learning, self-determination theory

## Abstract

The purpose of this research is to test the mediation effect of self-efficacy on college student's perception of teacher autonomy support and students' deep learning, and whether the peer support perceived by students can moderate the relationship between perceived teacher autonomy support and deep learning. A survey of 1,800 college students from a provincial undergraduate normal university in Guizhou Province in China was conducted through the revised Perceived Teacher Autonomy Support Scale, Deep Learning Scale, Self-Efficacy Scale, and Perceived Peer Support Scale (Mean age = 21 years old, SD = 1.34). Data use SPSS23.0, AMOS22.0 for descriptive analysis and correlation analysis, exploratory factor analysis (EFA) and confirmatory factor analysis (CFA), moderation effect, and mediation effect analysis. The research results show that after controlling for gender, major, and grade, self-efficacy partially moderates the connection between perceived teacher autonomy support and deep learning of college students. Moreover, perceived peer support mediates the relationship between perceived teacher autonomy support and students' self-efficacy.

## Introduction

Improving teaching quality is the core commission of higher education and the basic requirement of building a powerful country in education (Ine, 2021; Ruiz-Alfonso et al., 2021). Higher education is the principal force for cultivating talents in need of social development. In the current era of rapid development of information technology, deep learning ability represents the ability of innovation, creation and sustainable development, and is a crucial ability required under the background of current social and era development (Esteban-Guitart and Gee, 2020). Furthermore, the main teaching target person of higher education is undergraduates whose main duty at this stage is to learn how to learn, not to stay in the superficial understanding and mechanical memory of knowledge, but to understand in-depth knowledge, critically learn new knowledge, master knowledge through practical activities, exercise thinking, improve learning ability and innovation ability (Zhang et al., 2021). Therefore, the learning style of students is very crucial in the evaluation of teaching quality. More and more scholars are paying attention to let students learn from shallow learning to deep learning (Sølvik and Glenna, 2021). The concept of deep learning was first proposed by Marton and Saljo, who divided it into deep and shallow learning intentions and strategies according to students' reading methods (Marton and Säljö, 1976). They believed that deep learning is a learning method based on cognitive understanding and application and it's the primary strategy for students to meaningfully learn and understand from course materials and learning experiences (Marton and Säljö, 1976). Deep learning intentions and strategies form meaningful learning through deep processing of knowledge, forming a deep understanding of knowledge and a knowledge framework (Marton and Säljö, 1976). Shallow learning is the minimum effort for learning tasks and the acquisition of short-term memory of knowledge, while deep learning is the main strategy for the greatest investment in learning tasks and meaningful learning and understanding from course materials and learning experiences (Marton and Säljö, 1976). In the process of deep learning, students pay attention to the connection and structure of knowledge, achieve a deep understanding of problems and concepts, and obtain high-quality learning (Biggs, 1987; Marton and Saljo, 1997; Entwistle, 2001). Deep learning involves the brain's deep processing and understanding of knowledge, as well as the individual's subjective willingness to learn (Esteban-Guitart and Gee, 2020). It is an interdisciplinary study of neuroscience, psychology and pedagogy, and students with deep learning ability meet the educational requirements of system thinking and interdisciplinary sustainable development (Buckingham-Hatfield, 1996; Warburton, 2003), and the requirements for improving students' deep learning ability also meet the requirements of higher education for the quality of talent training (Filius et al., 2018). Moreover, the definition and connotation of deep learning have been deepening with the in-depth research of scholars. Such as Ryan and Deci (2000b) proposed that deep learning is a process of active learning, in which students have intrinsic motivation to learn, and their learning effect and academic performance will be improved to a certain extent. In the current research, the definition of deep learning mainly based on Biggs (1979), which means that students learn for understanding, mainly representing the critical understanding of the learning content, and highlighting the connection between prior knowledge and experience, and paying attention to logical relationships and evidence for conclusions.

Currently, teachers' autonomy support is widely regarded as one of the crucial exogenous factors in the research literature on the influencing factors of college students' deep learning (Kaplan, 2018), and teachers' autonomy support means that students get emotional identification from teachers and feel their support and encouragement for their autonomy decision and free choice (Ryan et al., 2014). In addition, it is found that teachers' autonomy support makes students feel more support and encouragement, which is not only conducive to the formation of a good teacher-student relationship but also promotes the deep learning style (Marshik et al., 2017). At the same time, it can also enrich students' self-efficacy (Ekatushabe, 2021).

However, learning is an active process for learners (Ryan and Deci, 2000a). When students think they have the ability to accomplish learning goals, their learning effect and performance will be enhanced (Ryan and Deci, 2000b). Previous studies have demonstrated that self-efficacy has a good positive predictive effect on students' deep learning style (Kuo et al., 2020). Self-efficacy is a form of individual thinking with oneself as the object which is an individual's belief, judgment or subject's self-perception of what level he or she can complete a behavioral activity before performing a certain behavioral operation (Bandura et al., 2001). Furthermore, self-efficacy has a direct impact on the performance of the individual's dynamic psychology in the implementation of learning activities and thereby has an impact on the actual learning activities (van Rooij et al., 2017), and with the improvement of self-efficacy, students' attention and executive ability have been improved (Elborolosy and Al Thenyan, 2020).

Students' self-efficacy was also influenced by relationships with others (Laird et al., 2018). When they receive recognition and support from others, they are more likely to have higher self-efficacy, while when trust and support from others are insufficient, psychological and behavioral problems may occur (van Rooij et al., 2017). Peers also play a crucial role in educational activities, the support of teachers to students and the support between students is of great importance to students (Schwab, 2019), therefore, the study of students' perceived peer support is also of great significance.

The current theoretical framework of research is mainly based on self-determination theory. Self-determination theory claims that self-determination experience is a core element of human motivation, goal pursuit, expressiveness, and perseverance (Deci and Ryan, 2000) and it asserts that human beings have three psychological needs: autonomy, relatedness, and competence, and when these three psychological needs are satisfied, creativity, motivation, and performance will flourish (Deci and Ryan, 2012). According to self-determination theory, autonomy needs are defined as control over processes and outcomes and strong intrinsic motivation, and the variables of perceived teacher autonomy support in the current study are proposed based on this psychological need (Deci and Ryan, 2000). Among these three demands, autonomy has the greatest impact on individual performance and expressiveness (Deci and Ryan, 2012). Relatedness is the need to establish an intimate relationship with others, to avoid the exclusion of the relationship, to establish a sense of belonging (Williams et al., 2005), therefore, the current research on perceived peer support is mainly based on this psychological need to verify whether peer support can provide the support of atmosphere and environment (Elliot and Church, 1997; Pintrich, 2000). Competence is a principle component of the motivational process of achievement, goal formation, approaching success and avoiding failure, and the self-efficacy variable in the current research are mainly proposed based on this psychological need (Núñez and León, 2015).

There is no research that explores the relationship between students' perceived teacher autonomy, perceived peer support, self-efficacy and deep learning. Therefore, the current research based on self-determination theory studies the effect of perceived teacher autonomy on students' deep learning and uses students' self-efficacy as a mediator variable to explore whether students' perceived teacher autonomy support can affect students' deep learning level by affecting students' sense of self-efficacy, and perceived peer support as the moderating variable to investigate whether peer relationship can moderate perceived teacher autonomy support and students' self-efficacy.

### Perceive Teachers' Autonomy Support and Students' Deep Learning

Autonomy support refers to the teaching method used by teachers to identify, train and establish students' intrinsic motivational resources (Reeve et al., 2004a). The behaviors supported by teachers' autonomy support include: providing the meaning of learning content, clarifying students' self-perception, using autonomy language, providing voluntary choices and cultivating students' internal incentive mechanism (Núñez and León, 2016). Specifically, teacher autonomy support is manifested in three aspects: organizational autonomy support which is mainly the comfort and happiness of the classroom environment, and program autonomy support which is mainly encouraging students to actively participate in classroom activities, and cognitive autonomy support which is mainly to encourage students to think about the content of learning at a deeper level and to have more lasting psychological engagement (Stefanou et al., 2004). In this atmosphere of autonomy support by teachers, students cannot feel compulsive teaching methods, their voluntary learning behaviors are encouraged, and follow their way to complete learning tasks (Ryan and Deci, 2004). According to self-determination theory, perceived teacher autonomy support refers to the degree of support or understanding of the student's understanding by the teachers (Mageau and Valler, 2003).

Student learning style refers to how students treat self-study and what strategies they adopt to treat self-study content (León et al., 2015). Deep learning means that students make meaningful connections between the content of learning materials and the original cognitive structure, and use deep learning strategies in the learning process. In contrast, shallow learning refers to students who use mechanical memorization of learning materials to satisfy basic course requirements (Marton and Säljö, 1976). Learning with deep or shallow learning methods is the result of the interaction between the student and the situation (Struyven et al., 2006). Studies have shown that using threats, deadlines, control evaluations, and tangible rewards that threaten students' perception of autonomy support will undermine students' learning and the degree of anxiety in the learning environment affects learners (Mouratidis et al., 2011). Students' academic performance in a learning environment that encourages autonomy discovery is better than their performance in a high anxiety environment (Ramsden and Entwistle, 1981). Oriol-Granado et al. (2017) demonstrated that students' positive emotions and autonomy support had a predictive effect on students' academic performance, self-efficacy and academic engagement, and Filippello et al., 2020) also demonstrated that teachers' autonomy support had an impact on students' academic performance.

Through searching the relevant literature on deep learning, there is no article to study the relationship between the perceived teacher autonomy support and students' deep learning. Therefore, based on the significance of filling the gaps in literature research and improving students' deep learning ability, and according to self-determination theory. Hypothesis 1 is proposed: perceived teacher autonomy support has a significant predictive effect on students' deep learning.

### The Mediating Effect of Self-Efficacy

According to self-determination theory (Ryan and Deci, 2000a), the key to students' learning motivation lies in satisfying three basic psychological needs, namely, competency needs, belonging needs and autonomous needs (Ryan and Deci, 2000a). The teacher actively understands the students' learning situation and ideas, gives full freedom and support in the selection of learning content, methods of solving problems, and minimizes the use of force and demanding methods in teaching (Deci and Ryan, 1985; Stefanou et al., 2004; Lam et al., 2009; Jang et al., 2010; Su and Reeve, 2011), these behaviors satisfy students' needs for competency and sense of belonging, and their psychological needs are met, which is conducive to the development of self-efficacy (Bandura et al., 2001). Self-efficacy is an individual's judgment and evaluation of the degree of completion before completing a specific task, and the degree of mastery of its ability to achieve goals (Bandura, 1997). If students feel a higher degree of autonomy support from teachers, they feel more external support (Fredricks et al., 2016), and satisfaction of psychological needs will lead to more interest in learning, initiative and a higher sense of self-efficacy (Hardre and Reeve, 2003; Chai et al., 2011; Cooper, 2014). Previous studies have proved that the perception of teacher autonomy support has a positive effect on self-efficacy, and teachers' provision of varying degrees of autonomy support can have an impact on students' learning process and results (Reeve et al., 2004b; Jang et al., 2010; Chen et al., 2015; Hospel and Galand, 2016; Sun, 2016; Zhang et al., 2018; Martin and Collie, 2019). Students who feel more self-supported by teachers are not only willing to complete the learning tasks assigned by the teachers independently, but also willing to accept more challenging learning tasks to prove themselves, thereby learning more knowledge and skills (Martin and Dowson, 2009).

The study by Bassi et al. (2007) and others explored the relationship between academic self-efficacy and deep learning, proved that if students have a lower academic self-efficacy, they will have a lower interest in learning and their learning style will be relatively shallow. The students with high academic self-efficacy are more interested in learning and are more willing to spend time and energy on learning (Ardura and Galán, 2019). Deep learning requires students to have intrinsic motivation for learning, and students with high academic self-efficacy are also one of the manifestations of high intrinsic motivation of students (Bandura, 1997; Marton and Saljo, 1997), and Oriol-Granado et al. (2017) demonstrated that students' self-efficacy can also predict higher levels of academic engagement. However, the research on perceived teacher autonomy support, self-efficacy, and deep learning is mostly limited to univariate or two-variable research, and there is a lack of research on the mechanism of mediation variables. Therefore, this article proposed hypothesis 2: self-efficacy plays the role of mediator between perceived teacher autonomy support and deep learning.

### The Moderating Effect of Perceived Peer Support

Peer support refers to the support and helps that students' perceived when they are studying, and whether it is practical activities in the classroom or academic tasks arranged by teachers outside the classroom, it needs the cooperation of peers to complete (Ladd, 1990, 1999). In this process, students' feeling the level of support is especially crucial for students' academics (Hofmann and Müller, 2018). Basis on self-determination theory (Ryan and Deci, 2000b), and relatedness obtain a sense of belonging and non-exclusion from peers and teachers in teaching scenes, and supports the relationship requirements of students (Williams et al., 2005).

Moreover, in the process of student development, peers are irreplaceable and indispensable, and peers play a vital role in the development of individuals (Hartup, 1982; Wentzel, 2005; Scholte and van Aken, 2006; Martin and Dowson, 2009; Parker et al., 2015). Young people meet the basic needs and development needs of their peers (Eccles et al., 1993; Deci and Ryan, 1995), especially the desire to connect with others or be recognized to support self-regulation (Brown, 2004). Moreover, some studies have shown that the welcoming attitude of peers can create a classroom atmosphere conducive to learning (Berndt, 2007; Gest et al., 2008; Kindermann and Skinner, 2012), and that peers are allowed to freely share their success, fear and concerns about school provide an emotional bond for peer support (Pekrun and Linnenbrink-Garcia, 2012). Furthermore, the values supported by peers and the feeling of being closely connected with peers are positively correlated with adolescents' learning interests (Wentzel et al., 2010) and learning motivation (Anderman and Anderman, 1999; Ryan, 2001; Nelson and DeBacker, 2008; Boud et al., 2014). At the same time (Harter et al., 1996; Hamm and Faircloth, 2005; Wentzel et al., 2010), also believe that peer support will affect students' motivational beliefs and emotional experience.

According to social cognition theory, the support of others, such as emotional encouragement, material help, and supportive information, which are individual feels, can enhance the individual's sense of self-efficacy (Bandura, 1997). Therefore, perceived peer support and perceived teacher autonomy support play a critical role in meeting the needs of relatedness.

The research on perceived teacher autonomy support, perceived peer support and self-efficacy is mostly limited to univariate or two-variable research, and there is a lack of research on the mechanism of moderation variables. Therefore, this article proposed hypothesis 3: perceived peer support plays the role of moderator between perceived teacher autonomy support and self-efficacy.

### Current Research

**Figure 1:**
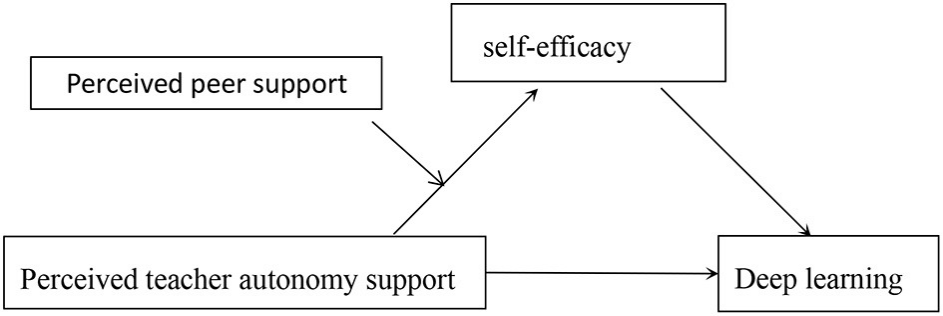
The proposed moderated mediation model.

The current research studied in a provincial undergraduate university in Guizhou province in China, and this study explored the relationship between students' perceived teacher autonomy support and perceived peer support, students' self-efficacy and deep learning. In this study, we have established a moderated mediation model to investigate the following hypotheses (Figure 1).

1. H1: perceived teacher autonomy support has a significant predictive effect on students' deep learning;
2. H2: self-efficacy can be a mediator between perceived teacher autonomy support and deep learning;
3. H3: perceived peer support can be a moderator between the perceived teacher autonomy support and self-efficacy.

## Method

### Participants

We carried out that study at a normal provincial undergraduate university in Guizhou, China. The university has a total of 13,559 full-time students and offers 46 undergraduate programs in 18 schools, covering 10 disciplines such as economics, law, education, literature, science, and engineering. One thousand eight hundred undergraduates (female = 1,300; male = 500) completed our study. The average age of the participants was 21 years (SD = 1.34). The students' subjects include science (29.7%), liberal arts (42.03%), engineering (9.80%), art (13.05%), and others (5.42%). Stratified sampling is adopted for the research objects, and the research objects are selected in different grades and majors. The 1,800 research objects can represent the general situation of the students of the school. Therefore, the data collected from the research objects can represent the validity of the research finding.

### Measures

#### Perceived Teacher Autonomy Support Scale

The perceived teacher autonomy support scale of this study was based on the Learning Climate Questionnaire (Núñez et al., 2012), adapted from the current teaching situation of the normal university in Guizhou province, China, and assessed students' perceived teacher autonomy support using 15 items. For example: I think most of my professional teachers provide a lot of autonomy activities; I think most of my professional teachers' pay attention to student-centered teaching when teaching. This scale mainly includes the organizational autonomy support, procedural autonomy support, and cognitive autonomy support of teachers to students. Each item uses a five-point scale ranging from 1 (complete non-conformance) to 5 (complete conformance). Pilot test (*n* = 40) assessed the validity and reliability of the scale, Confirmatory Factor Analysis (CFA) indicated a good model Fit, χ2/df = 4.779; RMSEA = 0.045; CFI = 0.973; TLI = 0.962; IFI = 0.973;RFI = 0.953; SRMR = 0.0294, with factor load ranging from 0.38 to 0.74, Cronbach's alpha indicated a high internal consistency of the Scale (=0.896), KMO = 0.935. This indicates that the reliability and validity of the scale are good.

#### Students' Deep Learning Scale

The deep learning scale of the students was the Revised Study Process Questionnaire (R-SPQ; Biggs et al., 2001), which is a modified version of the Study Process Questionnaire (SPQ); Based on Biggs, 1987 and based on the learning status of students in a normal university in Guizhou Province, China, he comprehensively selected 20 projects to evaluate the dimension of students' deep learning status. For example: I can synthesize and organize ideas, information, or experience to form new and more complex explanations and relationships; I can analyze the basic elements of ideas, experiences, or theories, such as an in-depth study of a specific topic and considering its components. Each item uses a 5-point scale ranging from 1 (complete non-conformance) to 5 (complete conformance). Pilot test (*n* = 40) assessed the validity and reliability of the scale, Confirmatory Factor Analysis (CFA) indicated a good model fit; χ2/df = 4.892; RMSEA = 0.046; CFI = 0.949; TLI = 0.941; IFI = 0.950; RFI = 0.927; SRMR = 0.0342, standardized factor load range from 0.43 to 0.72, Cronbach's alpha indicated a high internal consistency of the Scale (=0.936), KMO = 0.969. This indicates that the reliability and validity of the scale are good.

#### Self-Efficacy Scale

Based on the Academic Milestone self-efficacy Scale (Lent et al., 1986) and The Motivated Strategies for Learning Questionnaire (Pintrich et al., 1991), 5 items were selected to evaluate students' self-efficacy. For example: I believe that I can understand the complex knowledge taught by the teacher; I am confident that I can complete the homework and tests well. Each item uses a five-point scale ranging from 1 (complete non-conformance) to 5 (complete conformance). Pilot test (*n* = 40) assessed the validity and reliability of the scale, Confirmatory Factor Analysis (CFA) indicated a good model fit, χ2/df = 2.161; RMSEA = 0.025; CFI = 0.998; TLI = 0.995; IFI = 0.998; RFI = 0.990; SRMR = 0.104. The factor load range was 0.47 to 0.75, Cronbach's alpha indicated a high internal consistency of the Scale (=0.773), KMO = 0.811. This indicates that the reliability and validity of the scale are good.

#### Perceived Peer Support Scale

The Perceived Peer Support Scale in this study is based on The Classmate Support Scale (Torsheim et al., 2000), and comprehensively select 10 items to evaluate the peer support perceived by students in learning. For example: I like to study with other classmates or group discussions; I think group cooperative learning can enable me to master certain knowledge and skills faster. Each item uses a 5-point scale ranging from 1 (completely non-compliant) to 5 (completely qualified). Pilot test (*n* = 40) assessed the validity and reliability of the scale. Confirmatory factor analysis (CFA) indicated a good model fit, χ2/df = 1.226; RMSEA = 0.011; CFI = 0.999; TLI = 0.997; IFI = 0.986; RFI = 0.966; SRMR = 0.039. The standardization factor load range is between 0.42 and 0.71. Cronbach's alpha indicated a high internal consistency of the scale (α=0.730), KMO = 0.781. This indicates that the scale has good reliability and validity.

#### Control Variables

Previous studies have shown that gender, major, and grade are related to college students' learning engagement or performance (Jiang and Men, 2016). Therefore, in the following analysis, we control for gender, major, and grade level to avoid these control variables' influence.

### Procedure

The study was approved by the Academic Research Committee of the author's university. The researchers informed subjects were anonymous, and all subjects were willing to participate, and that they could withdraw from the study at any time. This study obtained the students' informed consent. The data collection process will be carried out by online software. Participants completed an online questionnaire, and well-trained teachers performed the data collection process.

### Data Analysis

SPSS version 22.0 was used for statistical analysis. Descriptive statistics were performed for all variables. We used the PROCESS of Hayes and Scharkow (2013) PROCESS Macro for SPSS (Model4) to further test the mediating effect of self-efficacy on SPSS. Finally, Hayes' PROCESS Macro for SPSS (Model7) was used to examine the moderated mediating effect of college students' perceived peer support on perceived teacher autonomy support and self-efficacy.

## Results

### Preliminary Analysis

The descriptive statistical results are shown in Table 1. The results showed that perceived teacher autonomy was positively correlated with deep learning (r = 0.84, *P* < 0.01) and students' self-efficacy (r = 0.72, *P* < 0.01). In addition, self-efficacy was positively correlated with college students' deep learning (r = 0.77, *P* < 0.01), and perceived peer support was positively correlated with college students' self-efficacy (r = 0.59, *P* < 0.01). Therefore, H1 is supported.

**Table 1:** Descripetive statistics and correlation among variables.

**Variables**	**M**	**SD**	**1**	**2**	**3**	**4**	**5**	**6**	**7**
1:Major	2.19	1.18	1						
2:Gender	1.72	0.45	0.05^*^	1					
3:Grade	1.80	0.98	−0.20^**^	−0.11^**^	1				
4:PTAS	3.43	0.60	0.03	−0.06^**^	0.02	1			
5:DL	3.51	0.55	−0.01	−0.03	0.03	0.84^**^	1		
6:SSE	3.22	0.67	0.00	−0.13^**^	0.07^**^	0.72^**^	0.78^**^	1	
7:PPS	3.59	0.59	−0.23	0.00	0.03	0.71^**^	0.78^**^	0.59^**^	1

*N = 1,832. PTAS, perceived teacher autonomy support; DL, deep learning; SSE, student self-efficacy; PPS, perceived peer support*.

* *P < 0.05*.

** *P < 0.01*.

### Mediating Effect Analysis

To test the mediating effect of self-efficacy on perceiving the autonomy support of teachers and deep learning of college students, we used Model 4 of the PROCESS Macro (Hayes and Scharkow, 2013) to estimate the three parameters. As shown in Table 2, in Model 1, perceived autonomy support of teachers has a significant impact on college students' deep learning (β = 0.76, *P* < 0.001). In Model 2, perceived teacher autonomy has a significant impact on college students' sense of self-efficacy (β = 0.56, *P* < 0.001). In Model 3, self-efficacy has a significant impact on college students' deep learning behavior (β = 0.52, *P* < 0.001). The perceived teacher autonomy support of teachers has a significant direct impact on college students' deep learning (β = 0.52, *P* < 0.001, SE = 0.015, 95% CI = [0.49, 0.55]), indicating that the sense of self-efficacy mediates the relationship between perceived autonomy support of teachers and deep learning of college students (AB = 0.25, SE = 0.015, 95% CI =[0.21, 0.27]). Mediating effect accounted for 31.81% of the total effect. Therefore, H2 is supported.

**Table 2:** Testing the mediation effect of perceived teacher autonomy support and students' deep learning.

**Predictors**	**Model1 (DL)**		**Model2 (SSE)**		**Model3 (DL)**	
	**β**	**t**	**β**	**t**	**β**	**t**
PTAS	0.76	65.74^***^	0.56	29.51^***^	0.52	35.31^***^
SSE					0.31	22.89^***^
R^2^	0.70		0.53		0.72	
F	1083.40^***^		509.29^***^		952.82^***^	

*N = 1832. Each column is a regression model that predicts the criterion at the top of the column. Covariates = gender, major, grade*.

*** *P < 0.001*.

### Moderated Mediation Effect Analysis

**Figure 2:**
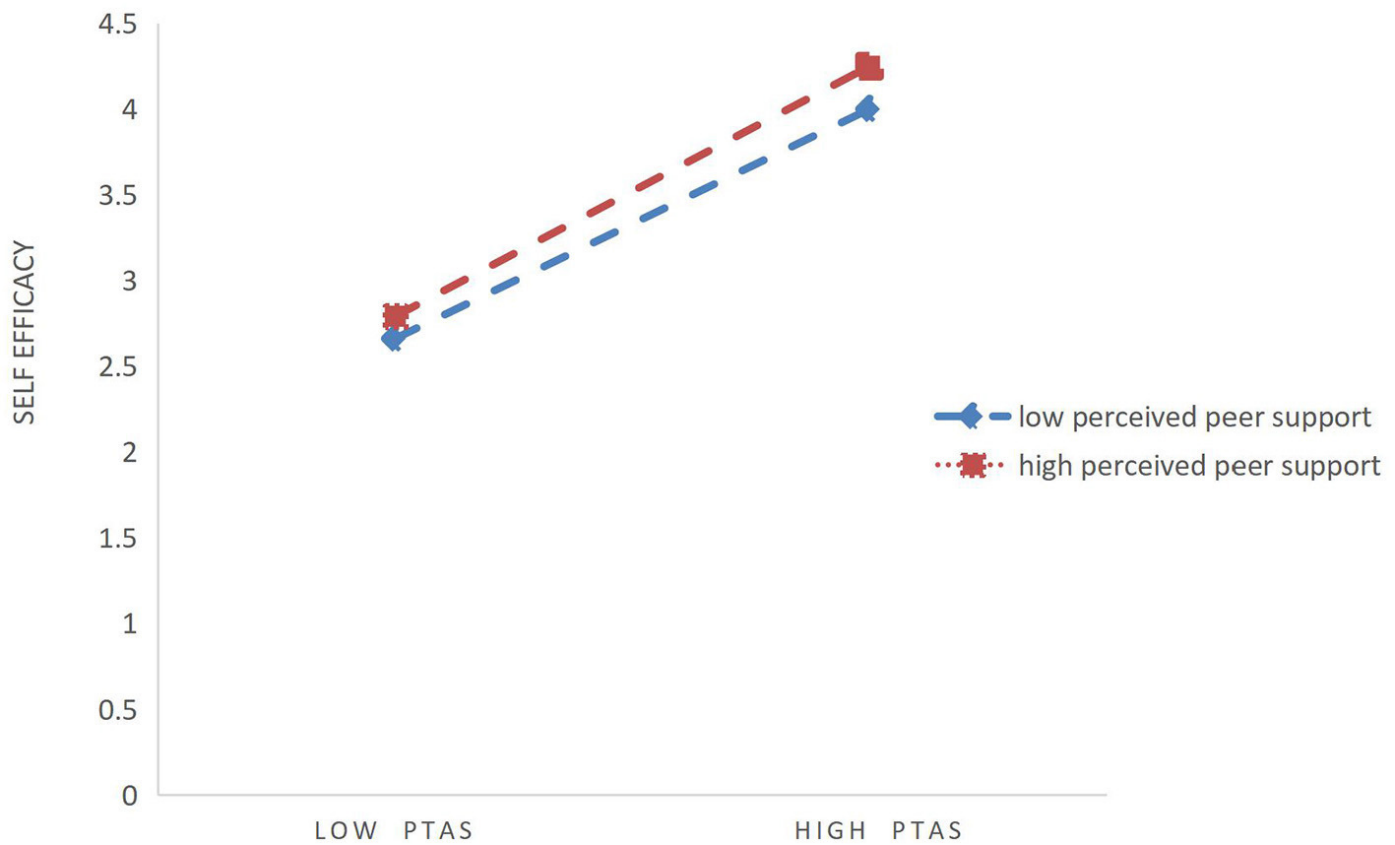
Interaction effect of perceived peer support and perceived teacher autonomy support on the self-efficacy. High and low levels of perceived support represent one standard deviation above and below the mean.

**Figure 3:**
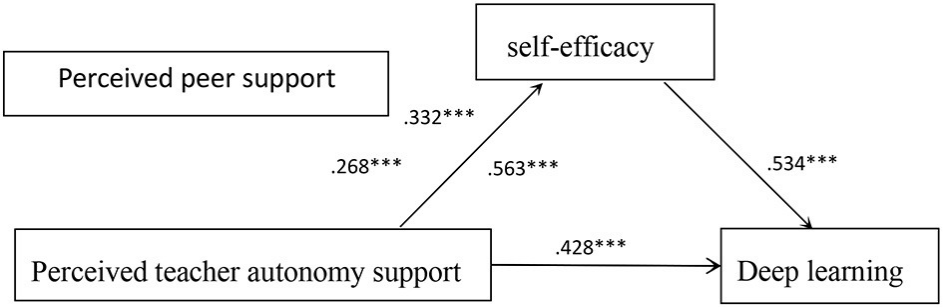
The confirmed moderated mediation model. ***p < 0.001.

H3 proposed that the peer support perceived by college students moderated the relationship between the teacher's autonomy support and self-efficacy. To test H3, Model 7 of the PROCESS (Hayes and Scharkow, 2013) macro was used. As shown in Table 3, the perceived teacher autonomy support has a significant impact on the self-efficacy of college students (β = 0.67, *P* < 0.001), and this relationship is moderated by perceived peer support (β = 0.58, *P* < 0.001). For ease of description, we used high peer support and low peer support (one standard deviation below the mean and one standard deviation above the mean) to draw a simple slope map of the prediction (Figure 2). A simple slope analysis shows that for the perceived peer support with a higher level, the high level of perceived teacher autonomy support is significantly related to the high level of student self-efficacy (βsimple = 0.71, *P* < 0.001). For low-level perceived peer support, low-level students' perceived teacher emotional support is significantly related to low-level students' self-efficacy (βsimple = 0.64, *P* < 0.001), and therefore support H3. Therefore, through the data analysis, the hypotheses were confirmed as show in Figure 3.

**Table 3:** Testing the moderated mediation effect of perceived teacher autonomy support and student deep learning.

**Predictors**	**Model1 (SSE)**		**Model2 (DL)**	
	**β**	**t**	**β**	**t**
PTAS	0.67	26.59^***^	0.52	35.31^***^
SSE			0.31	22.88^***^
PPS	0.19	7.27^***^		
PTAS × PPS	0.06	3.21^**^		
R^2^	0.54		0.77	
F	358.78^***^		1219.42^***^	

*N = 1832. Covariates = gender, major, grade*.

** *P < 0.01*.

*** *P < 0.001*.

## Discussion

### The Mediating Effect of Self-Efficacy

The results of the study illustrated that students' self-efficacy partially mediate between the perceived teacher autonomy support and the students' deep learning level. Therefore, this result confirmed self-determination theory (Deci and Ryan, 2000) in the current research which means when teachers use autonomy support teaching method, thereby students achieve more academic autonomy, and meet the basic competence psychological needs of students, and thereby students show stronger self-motivation and a sense of happiness, increasing students' self-efficacy and increasing students' competency psychology needs to obtain supportive deep learning behaviors (Cassidy and Eachus, 2000; Harris, 2003; Papinczak, 2009; Su and Reeve, 2011; Shen et al., 2016; Li et al., 2020). Moreover, the result demonstrated that perceived autonomy support can significantly predict deep learning. Therefore, the current research finding confirmed self-determination theory about the aspect of autonomy psychological needs and when the students perceived more organizational autonomy support, program autonomy support, and cognitive autonomy support, the students' engaged more in deep learning (Marshik et al., 2017). Therefore, the current research filled a gap in the literature research on the mediating role of self-efficacy between perceived teacher autonomy support and deep learning, which also laid a theoretical foundation for future research on the relationship between self-efficacy, perceived teacher autonomous support and deep learning.

In addition, research has proved that when teachers give students higher autonomy support, they can significantly predict students' self-efficacy. In other words, when teachers provide students with a relaxed, free, and autonomous learning environment, then students show higher academic confidence to achieve academic goals, and more confidence to solve problems encountered when encountering academic difficulties (Martin and Dowson, 2009; Geitz et al., 2016). In addition, the result explaining that for enhanced self-efficacy, teachers should improve students' self-efficacy from three levels: classroom environment, curriculum activities, and cognitive support, and further research should be conducted in this area including a more detailed classroom environment layout, classroom activity design and observation, and the relationship between cognitive knowledge and students' self-efficacy.

Research has also proved that when students have a higher sense of self-efficacy, they can significantly predict their deep learning level. Students have a higher sense of self-efficacy and will set a self-learning goal, and when students encounter academic difficulties, they will use a more active academic attitude to deal with academic challenges, actively seek solutions to problems, and actively seek solutions to problems in students, and maintain their learning status for a long time, and thereby when students run thinking and processing knowledge systems, deep learning will also produce (Chai et al., 2011; Cooper, 2014; Gutiérrez et al., 2021).

### The Moderating Effect of Perceived Peer Support

It is noted that the current research result confirmed that perceived peer support can moderate the relationship between perceived teacher autonomy support and self-efficacy. Therefore, this result supports self-determination theory (Deci and Ryan, 2000), specifically, perceived peer support meet psychological needs of relatedness which represents the relationship needs of the environment which is the main contextual for teachers, peers and students to interact (Reeve, 2009; Vansteenkiste et al., 2012; Moè et al., 2020). Moreover, perceived peer support could moderate the effect of perceived teacher autonomy on self-efficacy which broaden the research meaningful about the perceived peer support and self-efficacy relationship, thereby, the current research result indicated that relatedness psychological needs can have an impact on competence psychological needs (Oriol-Granado et al., 2017; Patall et al., 2018; Hall, 2019), and furthermore, among three basic psychological needs, autonomy and competence both have a significant effect on deep learning, and relatedness can moderate the effect of autonomy on competence needs. According to the results of moderating effect analysis, a higher level of relationship support, namely peer support, is more conducive to improving the influence of teachers' autonomy support on self-efficacy.

The current research result also supports the theory of social cognition,and when the environment satisfies students' emotional support, material assistance, and other supporting information, it can help students improve self-efficacy, thereby helping students improve their cognitive abilities in deep learning (Williams and Nida, 2011; Filippello et al., 2013, 2019, 2020; Alivernini et al., 2019). Moreover, the research fills a gap in the literature research on the relationship between perceived peer support, self-efficacy and perceived teacher autonomy support which also laid a theoretical foundation for future research.

### Limitations

The current research has several limitations to consider. Firstly, cross-sectional data do not allow causal inference due to the nature of cross-sectional research. Therefore, further experiments and longitudinal studies are needed to verify the research results. Furthermore, even though we control for potential confounding factors (gender, major, grade), there are still other confounding variables, such as general teacher emotional support that we cannot control. Additionally, the inadequacy of subjects' understanding of the definition of research variables has a certain impact on the research results. Lastly, the study sample was selected from one school, and the conclusions may be over-generation.

### Practical Significance

Despite the above limitations, this study has crucial theoretical and practical contributions. First of all, the current research is conducive to a deeper understanding of the mechanism of the perceived influence of teacher autonomy support on deep learning of college students. Moreover, the current research provides theoretical support for the future literature review on the relationship between perceived autonomy support, perceived peer support, self-efficacy and deep learning.

Secondly, through the survey, college teachers understand more about the current status of deep learning of students, and how to promote deep learning of students, increasing autonomy support of teachers, and contributing to the development of students' higher-order thinking ability (Marshik et al., 2017). In the context of teachers' autonomy support, students take the initiative to discover, explore and solve problems utilizing autonomy inquiry and peer cooperative learning. In the whole learning process, students' learning style is improved (Sølvik and Glenna, 2021).

Lastly, autonomy teaching contextual will be carried in the future practical teaching, therefore, the current research contributes to cultivate student self-awareness and initiative, and help students better acquire and transform knowledge, better transfer and use knowledge, cultivate higher-order thinking ability, and lay the foundation for training innovative talents (Sølvik and Glenna, 2021; Zhang and Yang, 2021).

## Conclusion

In general, the current research has established a moderated mediation model which can understand the influence mechanism between perceived teacher autonomy and college students' deep learning. The research results show that self-efficacy plays a mediating role between the perception of teacher autonomy and deep learning of college students. In addition, the mediation analysis shows that the perceived peer support mediates the relationship between perceived teacher autonomy support and college students' self-efficacy. According to this research conclusion, when students perceive more autonomy support and peer support, it is easier for them to carry out deep learning, and thereby students will improve their self-efficacy and enter the state of deep learning. Therefore, teachers should improve students' autonomy in practical teaching so that students can perceive more autonomy support, and on the basis of giving students autonomy support, teachers should strengthen organizational exchanges and mutual assistance and cooperation of students, and further enhance students' deep learning effects.

## Data Availability Statement

The raw data supporting the conclusions of this article will be made available by the authors, without undue reservation.

## Ethics Statement

The studies involving human participants were reviewed and approved by Guizhou Education University. Written informed consent to participate in this study was provided by the participants.

## Informed Consent

Informed consent was obtained from all individual participants included in the study, the research was also approved by the guardian of each participating student.

## Author Contributions

All authors are participants in the data collection and analysis and writing and revising the manuscript.

## Conflict of Interest

The authors declare that the research was conducted in the absence of any commercial or financial relationships that could be construed as a potential conflict of interest.
